# Complete Genome Sequence of Neonatal Clinical Group B Streptococcal Isolate CJB111

**DOI:** 10.1128/MRA.01268-20

**Published:** 2021-01-14

**Authors:** Brady L. Spencer, Anushila Chatterjee, Breck A. Duerkop, Carol J. Baker, Kelly S. Doran

**Affiliations:** a Department of Immunology and Microbiology, School of Medicine, University of Colorado-Anschutz Medical Center, Aurora, Colorado, USA; b Department of Pediatrics, McGovern Medical School, University of Texas Health Science Center, Houston, Texas, USA; c Children’s Memorial Hermann Hospital, Houston, Texas, USA; University of Maryland School of Medicine

## Abstract

Group B *Streptococcus* (GBS) is an asymptomatic colonizer of the female reproductive tract but can cause maternal and neonatal infections and adverse pregnancy outcomes. Here, we closed the genome sequence of strain CJB111, a neonatal GBS clinical isolate from a case of late-onset bacteremia without focus (Houston, TX; 1990).

## ANNOUNCEMENT

The Gram-positive beta-hemolytic bacterium Streptococcus agalactiae (group B *Streptococcus* [GBS]) asymptomatically colonizes the gastrointestinal and female genital tracts of healthy adults but can cause neonatal infections (pneumonia, bacteremia, meningitis [1]) and adverse pregnancy outcomes (2). Serotype V GBS isolates are emerging among adults and infants (3–6), and serotype V isolate CJB111 exhibits hypervirulence and vaginal persistence in murine models of GBS infection and colonization (7, 8).

CJB111 (ATCC BAA23) was isolated by Carol J. Baker from the blood of a female infant with late-onset sepsis on 18 July 1990, grown in Todd Hewitt broth (THB), and stored in glycerol at –90°C. The patient received intravenous ampicillin and gentamicin 1 day post onset of illness. Upon GBS isolation, therapy was switched on day 3 to intravenous penicillin (10-day treatment total). Following clean cerebrospinal fluid tests, she was diagnosed with bacteremia without focus at age 55 days and discharged without apparent sequelae. While CJB111’s sequence is currently available in 155 contigs (GenBank accession no. AAJQ01000000), a closed genome sequence may ease future genomic analyses.

CJB111 was grown statically overnight at 37°C in THB, genomic DNA was purified (Gentra PureGene Yeast/Bact kit), and concentration and quality were confirmed by NanoDrop spectroscopy. The Microbial Genome Sequencing Center (MiGS; Pittsburgh, PA) performed short- and long-read sequencing (Illumina and Oxford Nanopore technologies [ONT], respectively) and *de novo* assembly. Default parameters were used except where otherwise noted. Short reads were obtained using the Illumina Nextera kit and NextSeq 550 platform (9). For ONT sequencing, libraries were prepared using kit SQK-LSK109 to the manufacturer’s specifications (no DNA size selection/shearing), sequencing was performed on a MinION R9 flow cell, and base calling was performed using Guppy v4.2.2 (GPU mode) (10). Illumina paired-end reads (2 × 150 bp) and ONT long reads were provided as fastq files (Illumina: 7,410,044 reads, 989,364,400 bases, 472× coverage; ONT: 175,394 reads, 650,701,562 bases, 310× coverage, *N*_50_ value of 4,577 bp). bcl2fastq v2.20.0.422 was used for demultiplexing, quality control, and trimming of the Illumina reads (11) and Porechop v0.2.4 for quality trimming and removing adapters for ONT sequencing (12). Hybrid assembly via Unicycler v0.4.8 with a verbosity value of 2 (13) yielded six contigs, which were further assembled into three nonoverlapping contigs upon mapping to CJB111 contigs (AAJQ01000000) in Geneious v11.1.5 (14). The genome sequence was closed via PCR using primers flanking the nonoverlapping contigs (Table 1) and Phusion high-fidelity polymerase/buffer (New England Biolabs) under the following cycling conditions on a Bio-Rad T100 thermal cycler: 98°C, 2-min hot start; 34 cycles (98°C, 10^seconds^; *T_m_* °C, 20^seconds^; 72°C, 30- second extension/kb); and 72°C, 10-min extension. Purified PCR amplicons (Qiagen) were Sanger sequenced using Applied Biosystems 3730/3500xl genetic analyzers, yielding 2× sequencing in both directions. Contigs were assembled *de novo*, overlapping ends were trimmed, and the genome sequence was rotated manually in Geneious v11.1.5 to start with *dnaA*.

**TABLE 1:** Primers used in this study

PCRs flanking nonoverlapping contigs	Primer name^*a*^	Sequence (5′–3′)	PCR *T_m_* (°C)	PCR product length (bp)	PCR extension time (min:s)
PCR 1	5121	TATCAATAACGATAGTATGCCCAGCG	65	4,712	2:21
	3121	TTCCAATAGGTCTTGATAGTGAGGTG			
PCR 2	5122	GTTTGTTGCAGTCGTCGTTATCTC	63	3,392	1:42
	3122	CGTCGGAATTAAATCTTGGAATACC			
PCR 3	5123	GGCATCAGGAATGATCTGATTTACAC	65	2,064	1:02
	3123	TGCCTCCCATTGGATTACTGTATAC			
PCR 4	5124	GACTCGATAGGGTATATGGTGCC	65	5,126	2:34
	3124	GGTTCGATTGCGTTACTGCG			
Sequencing primers					
PCR 1	5131	GTGACATAGATTGGAATAGGGTTAGC			
	5132	TATTCTCAGTGTCTGTGTACTACTGC			
	5133	AAATCTTGGCAGACAGTGGTTATC			
	5134	CAACAGGAGGAACCTGTAGAAGTTC			
	5135	TACAATCCATCTCTGGAATTCAC			
	3131	GTGACATAGATTGGAATAGGGTTAGC			
	3132	ATAATAAGGTGTCAGACAAACTCGC			
	3133	GGTTCGTCATTTATGAATGGTGTAC			
	3134	TTGACTATGGTTATGCTTTCAGG			
	3135	TTCTCAACCTTGATTCTCTCTTTGG			
	3136	GTGCCGTTTCAAAGGTCGCT			
	3137	CCGGGCTCGCTCCATATAGATAAG			
PCR 2	5136	TATGCTCTCATAGGTAACACCACC			
	5137	AACGATCACCTAAATTAGTACCTGC			
	5138	TCTATCTTGTTCCTGTTTCCTTG			
	5139	TTTAGGTTAGAAAGGAGATACTGCC			
	5140	TACTTCAAATGGTATGCAAGCTATGG			
	3138	GCTGAACAAGCTGCTGTTATTGC			
	3139	TTTAGTTGAGGATGCTTATCGAG			
	3140	AGTTATCTGTCTATAAGGAATGTCG			
	3141	AAGCTATGGTTGAAGCTGTTG			
PCR 3	5141	TTAAATTAACTCCTGAAGTACTCCG			
	3142	AGGTAATTTCCATTTCTCACCTGAAG			
	3143	TTTCGGCGACAATTCATTGAACTGAG			
PCR 4	5125	GTAACTAGTTATCTCTAGCCTGTAGC			
	5126	CACGAAAGCAACTTAATCCGTCG			
	5127	CCCTTGACTACATAAGTACTAACCC			
	5128	CTGTTAATAAATCAGCTCCATGAGC			
	5129	TTTCCCTTGCATTTCCCATAGACC			
	5130	GCCTATCCAATTATTCGTTTGGAG			
	3125	TTTACCTCTGTTGCATCCACAATC			
	3126	GCAAAGCAATTGTATTCCGTCTT			
	3127	AAAGTGTCGTTACCAACTCTGAAG			
	3128	AAATTATGAATCAGGCATGCTCCTGG			
	3129	AATAAAGCCTGAAACCAGTTCAGAG			
	3130	CATCACTCTGGCCTCTATTATTT			

a Primer names beginning with 5 indicate forward primers. Primer names beginning with 3 indicate reverse primers.

The CJB111 sequence was deposited at GenBank as one circular contig (2,093,987 bp; GC content, 35.52%). BUSCO_v1 and CheckM v1.0.18 confirmed the genome completeness (15, 16). GenBank annotated the CJB111 genome sequence using PGAP v4.13 (17).

### Data availability.

The CJB111 sequence is available in GenBank under accession no. CP063198. The raw sequence reads are accessible under Sequence Read Archive accession no. SRX9273111, SRX9273112, and SRX9273113; BioProject accession no. PRJNA663970; and BioSample accession no. SAMN16191206.
